# Effect of a peer‐led intervention combining mental health promotion with coping‐strategy‐based workshops on mental health awareness, help‐seeking behavior, and wellbeing among university students in Hong Kong

**DOI:** 10.1186/s13033-020-00432-0

**Published:** 2021-01-09

**Authors:** Daniel Kwasi Ahorsu, Dalinda Isabel Sánchez Vidaña, Donald Lipardo, Parth Bharat Shah, Pablo Cruz González, Sachin Shende, Shilpa Gurung, Harun Venkatesan, Anchalee Duongthipthewa, Talha Qasim Ansari, Veronika Schoeb

**Affiliations:** 1 Department of Rehabilitation Sciences, The Hong Kong Polytechnic University, Hong Kong, SAR China; 2 College of Rehabilitation Sciences, University of Santo Tomas, Manila, Philippines; 3 School of Design, The Hong Kong Polytechnic University, Hong Kong, SAR China; 4 Department of Civil and Environmental Engineering, The Hong Kong Polytechnic University, Hong Kong, SAR China; 5 Department of Applied Biology and Chemical Technology, The Hong Kong Polytechnic University, Hong Kong, SAR China; 6 Institute of Textiles and Clothing, The Hong Kong Polytechnic University, Hong Kong, SAR China; 7 Department of Mechanical Engineering, The Hong Kong Polytechnic University, Hong Kong, SAR China; 8 School of Health Sciences (HESAV), University of Applied Sciences and Arts Western Switzerland (HES-SO), Lausanne, Switzerland

**Keywords:** Mental health, Help‐seeking behavior, Mental health promotion, Coping skills, University students

## Abstract

**Background:**

The psychological well-being of university students is an important factor in successfully coping with the demands of academic life. This study aimed to assess the impact of a peer-led intervention of mental health promotion combined with coping-strategy-based group workshops on mental health awareness and help-seeking behavior among university students in Hong Kong.

**Method:**

A mixed-method concurrent design was used for this study. Quantitative data, based on one-group pretest-posttest design, were collected using Mental Health Knowledge Schedule Questionnaire to assess mental health awareness, and Attitude Towards Seeking Professional Help Questionnaire-Short Form to examine help-seeking behavior of university students from The Hong Kong Polytechnic University. Qualitative data were collected from written post-activity reflections and focus group discussions which were thematically analyzed.

**Results:**

A total of 62 university students (mean age: 23.2 ± 5.1 years) were included in this study. Mental health awareness was significantly improved (*p* = 0.015, 95% Confidence Interval of − 2.670, − 0.297) after program implementation. Help-seeking behavior mean score increased from pretest to posttest, however, no significant difference was observed (*p* = 0.188, 95% CI = − 1.775, 0.355). Qualitative analysis revealed that the program helped participants learn about coping strategies to help themselves and others with mental health challenges.

**Conclusions:**

The peer-led intervention provided a positive impact through increased mental health awareness and knowledge of coping strategies on self-help and helping others among university students. Further study could focus on the impact of the program when applied regularly throughout the entire academic year.

## Background

Myths, stereotypes, stigma and prejudice concerning mental illness are serious healthcare issues affecting persons with mental health problems that hinder them from seeking professional help [1]. The onset of mental illness in 75% of cases is between 17 and 24 years [2], a time when students enter higher education suggesting higher vulnerability to mental health challenges [3]. Students face enormous stress that cuts across continuing pressure from academics and peers to conform, and from family members to excel, often with minimal and/or maladaptive coping strategies resulting in developing mental health problems [2, 4].

Mental health promotion contributes to enhancing an individual’s ability to reach and maintain a positive psychosocial state that helps to cope with daily life adversities [5]. It also helps in strengthening community assets to help prevent mental disorders apart from enhancing the well-being and quality of life of community members [6]. Hence, educating university students on mental health issues would equip them to identify, assist in the management and prevention of mental health issues, as well as help mitigate their negative attitude towards mental health issues, assisting in educating their peers on mental health issues and how to seek help,

Counselling centers in academic institutions are readily available to provide services to help students with mental illness, however, the stigma attached to consulting school counselors deter students from accessing these services [7]. Help-seeking behavior remains one of the main bottlenecks in mental health due to factors such as stigma, embarrassment, and problems recognizing symptoms [8, 9]. Help-seeking behavior refers to a complex decision-making process that is triggered by a problem that challenges the capabilities of an individual [10]. The main attributes of this process include problem focused, intentional action, and interpersonal interaction. Regarding health-related issues, help-seeking behavior refers to planned behavior focused on a health-related issue that involves interpersonal interaction with a chosen health-care professional [10]. Innovative strategies must, therefore, be used to break this menace when seeking help for mental health concerns and should be the least humiliating possible [9]. Strategies for promoting mental health and help-seeking behavior include a wide range of activities at community-level intervention (social policy), organization-level intervention (e.g. workshops at institutions), and individual-level interventions (e.g., psychotherapy) [11].

The social component in mental health promotion strategies is important to enhance the sense of care, support, and belongingness [11]. Therefore, strategies which include group interaction may be used as it may add a positive social effect on the effectiveness of mental health promotion. We hypothesized that the combination of mental health promotion with group activities that focused on the improvement of wellbeing would act in synergy to improve students’ knowledge on mental health and motivate their help-seeking behavior [12]. In addition, experiencing the benefits of different physical activities or relaxation techniques, whose settings were designed and focused on mental health, would allow the participants to learn and experience ways to tackle emotional distress and manage their emotions. Therefore, the present study examined the effects of a peer-led intervention of mental health promotion combined with coping-strategy-based group workshops on mental health awareness, help-seeking behavior, and wellbeing among university students. Specifically, the study (1) examined the effect of a mental health promotion on mental health awareness and help-seeking behavior (quantitative study); and (2) explored the perceived impact of the intervention (mental health promotion and workshops) on participants’ mood (mental health state) and well-being (qualitative study).

## Methods

### Research study as part of a student‐initiated suicide prevention project

The current study was an offshoot of a student-initiated youth suicide prevention project called “Game of Tones” which was sponsored by the WeCare Fund of The Hong Kong Jockey Club Centre for Suicide Research and Prevention of the University of Hong Kong. The aim of WeCare fund projects was to encourage students to initiate, plan, organize, and implement a mental health related projects for their peers to participate at their own campuses. The peer-led term is used in the present study because the authors who implemented the project and the target population of the project were students from the same institution which was a condition of the WeCare Fund. The “Game of Tones” Project was made open to any bona fide university student of The Hong Kong Polytechnic University. Invitations to participate were sent via email, disseminated using brochures and promoted using posters paste on campus and student hall bulletin boards.

### Intervention: project “Game of Tones”

The intervention implemented from the “Game of Tones” Project comprised two core components: (1) mental health promotion (video and booklet) and (2) coping-strategy-based group workshops. The rationale behind this combination approach was not only to transmit knowledge on mental health (facts and myths of mental health) to improve their mental health awareness, and highlight the importance of seeking help, but also to provide participants with an appropriate way of relaxing or de-stressing to improve their help-seeking behavior and wellness [7]. This combined intervention approach is consistent with the objectives of the study.

The mental health promotion consisted of a 7-min video and a booklet. The video and booklet provided information on mental health such as the definition of mental health, myths and facts about mental illness, examples of famous people with mental illness and their contribution to society, strategies for self-help and promoting help-seeking when emotionally distressed, and strategies to help others. Additionally, information on the benefits of the activities on mental health, contact information of counselling centers at the hospitals, on campus, and counselling and suicide prevention hotlines were included in the booklet. The same video was repeatedly presented prior to starting any workshop, and the booklet was provided at the conclusion of every workshop.

The first set of workshops focused on mindfulness, relaxation activities and internalized work where the participant had a passive role [13–15] which was termed *outside-in approach*. The second set of workshops included dynamic activities and externalized work where participants had an active role in de-stressing which was termed *inside-out approach*. The *outside-in activities* included (1) progressive muscle relaxation (PMR), (2) Vipassana meditation, and (3) Yoga while the *inside-out activities* included (1) kickboxing and (2) fitness. These coping-strategy-based workshops were carefully selected based on reported beneficial effects on mental health. PMR is an anxiety-reduction technique [16] involving alternating tension and relaxation in all the body’s major muscle groups. By practicing PMR, it is possible to reach a sense of control over the body in situations when muscles are tense [15]. Vipassana meditation is a technique guiding a person to achieve peace of mind and is described as a logical process of mental purification through self-observation. Practicing Vipassana meditation on a regular basis strengthens the inner self to face everyday difficulties with stronger motivation, effort, patience, and contentment [17]. Yoga is an ancient Indian technique of unifying the mind, body and soul that evokes a calming effect [18]. The practice of yoga has shown to help students get into a frame of mind conducive to learning and distinctive from the effects of physical exercise alone [13]. The fitness and kickboxing activities focused on exercises to release stress, improve mood, and increase self-esteem [19, 20]. Furthermore, physical exercise has been shown to decrease tension and reduce depression and anxiety [21, 22].

All the workshops were conducted by invited experts and designed for novice or beginners level participants. For instance, the intensity of the fitness and kickboxing workshops was low and the meditation, yoga and PMR workshops covered the basics of the techniques. The duration of the whole session including the completion of the questionnaires, viewing of mental health promotion video, and participation in a workshop varied depending on the nature of the workshops. The duration was 3 h for the fitness and kickboxing workshops, 1.5 h for the PMR and meditation workshops, and 2.5 h for the yoga workshop. Participants were free to choose the workshop of his/her preference and were permitted to participate in several group workshops, however, only those who fulfilled the selection criteria were included in the quantitative and qualitative data analysis. At the end of each workshop, aside from receiving the program booklet, participants were instructed to provide immediate post-activity feedback.

### Study design and setting

**Fig. 1:**
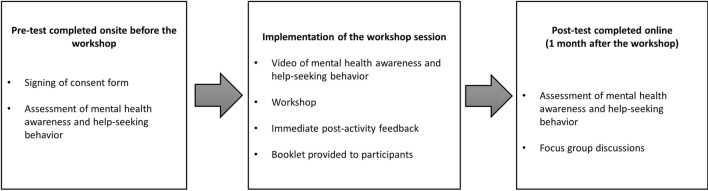
Overview of the program implementation and data collection

A mixed-method concurrent design was used to attain a comprehensive understanding of the effectiveness and impact of the peer-led intervention consisting of a mental health promotion combined with group workshops [23]. Ethical approval for this study was obtained from The Hong Kong Polytechnic University (IRB HSEARS20170921002). The quantitative and qualitative methods were used to collect the data concurrently, but analyzed separately in accordance with the rules of each method [24]. The quantitative component relied on a one-group pretest-posttest design, while the qualitative component used post-activity written reflections and focus group discussions. The intervention, assessments, and focus group discussions were conducted in English at The Hong Kong Polytechnic University from September 2016 to October 2017. Figure 1 shows an overview on how the study was conducted.

### Study participants

The study participants were conveniently recruited from the set of undergraduate, graduate and postgraduate students of The Hong Kong Polytechnic University who took part in our “Game of Tones” project. They were included in the quantitative part of the study if they engaged in one only interventional session (i.e., a mental health promotion combined with a group workshop) and if they gave consent for their data to be included in the analysis. They were excluded if they joined multiple activities because they would have listened to and participated in the activities several times which can potentially affect their responses to the questionnaires. Data for the qualitative part of the study came from two sources. The first source was obtained from the immediate post-activity feedback from “Game of Tones” participants who joined in the mental health promotion session and at least one workshop, whether or not they completed the post-test questionnaires after one month. The second source of data were gathered from the focused group discussions involving those who joined the mental health promotion and at least one workshop, and who also completed the post-test questionnaires after one month.

### Measures for the quantitative part of the study

All participants answered three self-administered questionnaires onsite just prior to watching the mental health promotion material (video) and participating in a workshop. The first questionnaire collected socio-demographic information such as age, sex, educational level, nationality, and email address. The other two questionnaires were the Mental Health Knowledge Schedule Questionnaire (MAKS) [25] to assess their mental health awareness, and the Attitude Towards Seeking Professional Help Questionnaire-Short Form (ATSPPH-SF) [26] to examine help-seeking behavior. The MAKS is a short 12-item tool for assessing and tracking mental health-related knowledge which was developed by Evans-Lacko et al. (2010) [25]. It has a moderate to substantial internal reliability and test–retest reliability. The scoring system is based on a 5-point Likert scale (1 = strongly disagree to 5 = strongly agree) [25]. The total score is obtained by adding the points from the 12 items. Higher total scores mean greater mental health awareness [25]. The ATSPPH-SF is a 10-item scale on attitudes toward seeking professional help developed by Fisher and Farina (1995) [26]. It has a four-point Likert-scale response format ranging from Agree (3), Partly Agree (2), Partly Disagree (1), and Disagree (0) [26]. The responses are summed up (items 2, 4, 8, 9, and 10 are reverse scored) to get a total score with higher scores indicative of positive awareness of help-seeking behavior [26]. The participants were followed-up via email one-month after their participation in order to gather the post-test online responses to the MAKS and ATSPPH-SF questionnaires.

### Qualitative data

At the end of the intervention session, participants were requested to complete an immediate post-activity feedback form where written reflections were obtained. Participants were invited to write openly, anonymously or not, on stationery and on a post-activity feedback form about their perceptions/feelings before and after the intervention as well as sharing comments related to the intervention. Participants who completed the post-test assessments were invited to participate in the focus group discussions. Two focus group (FG) discussions were conducted at the end of the program with 17 participants (FG 1: N = 10; FG 2: N = 7). The interview guide included questions regarding participants’ understanding of mental health and on the effects and impact of the mental health promotion with the group workshops on their mood and well-being. The following questions are examples of the questions used in the focus group discussions: *“What do you consider as mental health challenges?”* or *“How could or would you act in situations of mental health challenges?”* as well as questions such *as “Did your perceptions about mental health issues change compared to before the activities?”* Each focus group lasted for about 60 min. Transcription of the discussions was performed separately by two researchers (DISV and DL) and validated by another researcher (VS) before analysis.

### Data analysis

The quantitative data analysis in combination with qualitative data extracted from the immediate post-activity reflection and the focus group discussions were carried out to ensure comprehensiveness and triangulation of results. Descriptive statistics was used to summarize demographic data and paired t-test was utilized to assess changes in the scores before and after the program (α < 0.05). Quantitative data analysis was conducted using IBM SPSS version 23 for Windows. For the qualitative data, thematic analysis [27] of all transcripts from the immediate post-activity feedback and focus group discussions was performed by three researchers (DISV, DL, and PCG). Consolidation and summary of codes, categories and themes were performed by another researcher (VS) who is an expert in qualitative analysis.

## Results

### Quantitative data

**Fig. 2:**
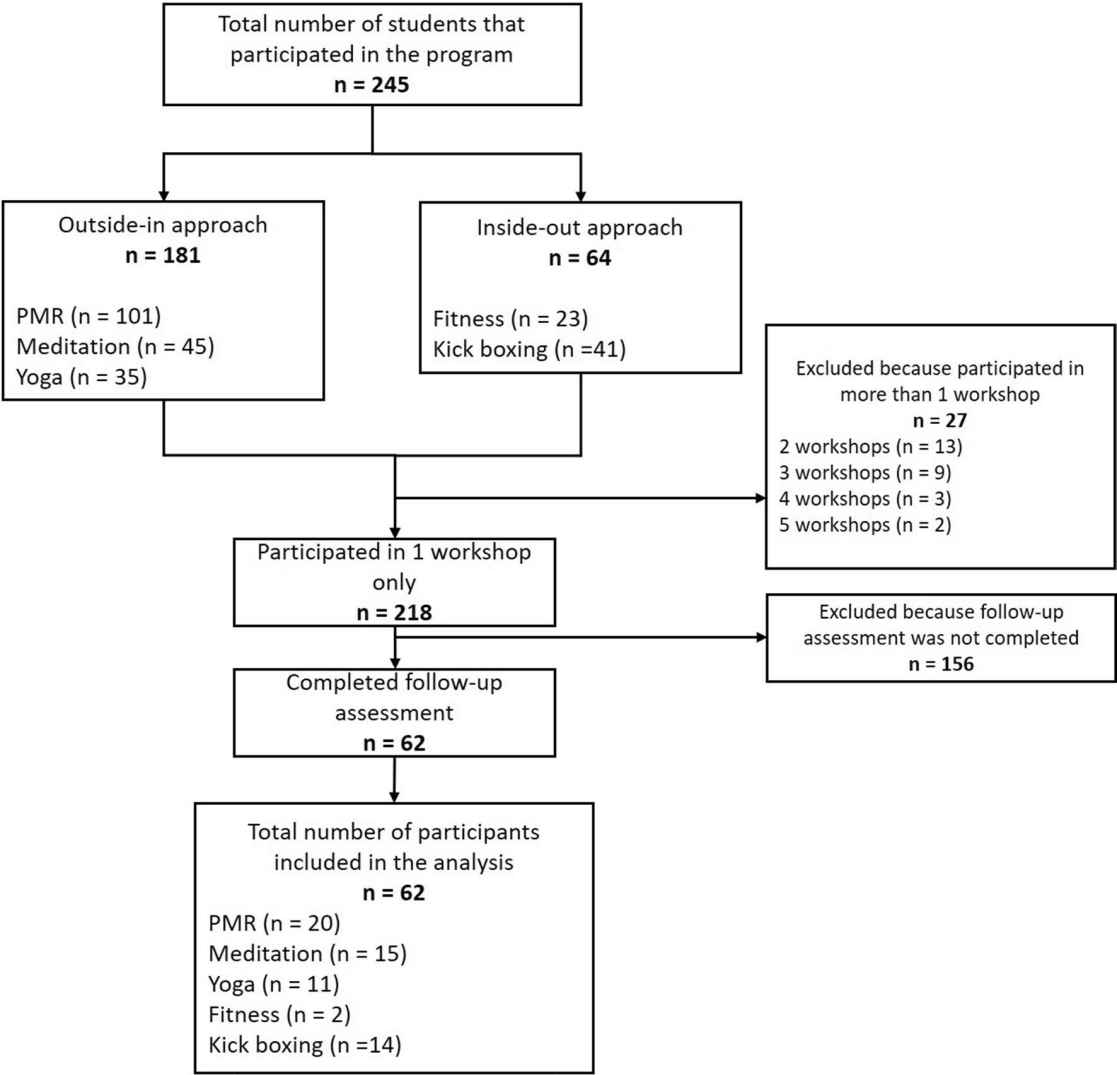
Flow of participants included in the quantitative analysis

From a total of 245 individuals who participated in the “Game of Tones” Project, 62 students were found eligible and included in the quantitative component of the study (25% of participants in one workshop only). Figure 2 shows the consort flow diagram of the study. Table 1 presents the summary of demographic background of all the participants included in this study.

Table 2 shows the pretest and posttest mean scores and standard deviations of the participants based on the two questionnaires. For the awareness of mental health, there was a statistically significant increase in mean scores (*p* = 0.015 with 95% confidence interval of − 2.670, − 0.297) from pretest to posttest. Higher total scores mean greater mental health awareness. However, for awareness of help-seeking behavior, while the mean score increased from pretest to posttest, the difference was not statistically significant (*p* = 0.188, 95% CI = − 1.775, 0.355)

**Table 1:** Demographic characteristics of the participants (n = 62)

Variable	Frequency	Percentage (%)
Age (years)		
17–20	26	41.9
21–30	32	51.6
31–38	4	6.5
Sex		
Male	17	27.4
Female	45	72.6
Country of Origin		
Local (Hong Kong students)	8	12.9
Non-Local (International students)	54	87.1

**Table 2:** Pretest and posttest scores on mental health awareness and help-seeking behavior

	Pretest	Posttest	*p*	95%CI
Awareness of mental health, $\underline{x}$±SD	40.55 ± 3.94	42.03 ± 3.70	0.015	-2.670, -0.297
Awareness of help-seeking behavior, $\underline{x}$±SD	21.47 ± 3.51	22.18 ± 3.50	0.188	-1.775, 0.355

$\underline{x}$ mean; *SD* standard deviation; *CI* confidence interval

### Qualitative data

The thematic analysis of the post-activity reflections and focus group discussion transcripts revealed five themes: (1) definition and attitudes of mental health, (2) challenges when dealing with mental health, (3) strategies to deal with mental health challenges, (4) strategies to help others, and (5) positive impact of the program. Table 3 presents the Themes, categories and codes generated.

**Table 3:** Results of the thematic analysis of focus group discussions presenting codes, categories and themes

Themes	Categories	Examples of codes included per category
Definition and attitudes of mental health	Relaxation	Being relaxed; Sleep well to do other activities
	Emotions	State of mind, feeling down, depressed, stressed out
	Control	Ability to deal with emotions and manage stress
	Loneliness	Being alone is difficult
	Positive thinking and resilience	Positive attitude to life; Never give up when you are depressed
	Confidence	Believe in oneself and be confident
	Elements needed for good mental health	Different attitudes to deal with stress
	Helpful actions	Exercise to release stress or anger; Getting distracted from the problems
Challenges when dealing with mental health	Signs of emotional distress	Seeing only the negative; Struggle to control
	Negative impact of mental health problems	It affects social life; Difficulty to manage peer and academic pressure
	Boundary between normal state and abnormal emotional state where help is needed	Difficult to differentiate whether improving or worsening
	Stigmas associated to mental illness	People are fearful of mental health problems; Recovery is not possible
Strategies to deal with mental health challenges	Communication and sharing	Express and not keep the problems to oneself; Talk to friends and close people
	Self-care	Relax to find inner peace
	Being active	Get involved in activities; Physical activity is helpful
	Spiritual belief	Praying to God helps
	Seek professional help	Professional help is important Professional help may lead to faster recovery
Strategies to help others	Communication and sharing	Listening and sharing similar experiences
	Feedback and advice	Provide positive feedback
	Social support	Social support, stress relief together
	Seek professional help	Seek professional help
Positive impact of the program	Understanding of mental health	Mental health problems are quite normal; Willingness to seek professional help
	Good and helpful	Learned how to get out of negative issues; A way to break the routine, relax and not to think; Help improved sleep; Taking care of oneself; Strategies to improve mental health
	Extended impact (using what was learned to help others)	Sharing information from the program to help others

**Theme 1: Definition and attitudes of mental health.**

Describing the views of some participants, they mentioned that mental health is defined as having the ability to find balance in life by being in control over pressure, stress, mood, and depression. The information in theme 1 showed the knowledge that most of the participants had on mental health which is relevant to explore mental health awareness.

P2: "*Mental health is the ability to control and find balance between good and bad moods*."

On the other hand, some participants indicated that those with mental illness or disturbance show loneliness, depression, feel down and stressed out which may not be readily noticeable because they seem to look good despite of having these problems. The information shared by the majority of the participants on the signs associated to poor mental health suggests knowledge on mental health and serves as an indication of mental health awareness.

P11: “*With mental health, one could only see the negative and will find it hard to see the positive*.”

**Theme 2: Challenges when dealing with mental health**.

Participants’ challenges when dealing with mental health were reported to be signs of emotional distress (struggle to control, feeling that there is no way out), the negative impact of mental health problems (they affect their studies and social life), stigmas associated to mental illness (e.g. people are fearful of mental health problems, recovery is not possible) and the difficulty to distinguish the boundary between a normal and atypical emotional state where help is needed. In order to evaluate help-seeking behavior, it was crucial to explore the participant’s views on mental health challenges. In this theme, some participants described characteristic signs of mental health challenges. Recognition of mental health challenges is relevant as part of the process towards help-seeking.

P3: “*One challenge is the other people’s thoughts about someone having, coping and dealing with mental health problems, being treated like a patient or something.*”

**Theme 3: Strategies to deal with mental health challenges.**

Participants’ dealing with mental health challenges included communication and sharing (talking to friends), self-care activities such as sleep, “slowing down” and getting relaxed, being active (e.g. physical activity), having spiritual beliefs, and seeking professional help leading to a faster recovery. The participants’ views on strategies to deal with mental health challenges provides information on their knowledge of support strategies which is relevant for the assessment of help-seeking behavior.

P10: “*To get rid of mental sickness, you can interact with people, be fit, meditate, watch movies, roam around, and take a short break from work, particularly those involved in research*”.

**Theme 4: Strategies to help others.**

Some participants also expressed ways on how they can reach out for and help those who suffer from mental illness. The strategies include listening to the person that needs support, giving feedback and advice, offering social support so that the person is not alone and referral to qualified professionals. Although this theme is out of the scope of the research questions, the information regarding helping others suggests knowledge of coping strategies that could be associated to what was learned in the intervention.

P8: “*If listening doesn’t help the person facing challenges, seeing a medical practitioner would be the best because they have other ways to handle the issues.”*

**Theme 5: Positive impact of the program.**

The program was well-received and had a positive impact on participants’ views and understanding of mental health issues. Some students mentioned that they became aware that experiencing mental health problem is normal. Also, they mentioned that joining the activities (mental health promotion + workshops) was a way to care for oneself and became willing to seek professional help as a part of a self-care strategy.

P2: “*The workshop helped me understand that having mental health problems is quite normal to people. It helped me become more willing to seek professional help to overcome problems*.”

Some students found the workshops to be helpful. They attested that the activities helped them to release energy and negativity, and to get relaxed. Furthermore, participants shared that they acquired new skills and knowledge by joining the activities which is relevant to assess their perceived impact of the intervention.

Finally, the positive value of the activities (mental health promotion + workshops) was also reflected in the participants sharing the information learned in the workshops with family and friends which could be considered as potential extended impact of the activities. The participants appreciated being able to use and share the information learned from the intervention to help others. One participant introduced meditation to her mother that helped her to relax from work. They would like to recommend it to be a continuous program offered to university students, some even suggesting that the intervention “*to be included in the coursework so that (more) people can come” (P2).* The participants mentioned that they shared information from the activities with family and friends, but the extended impact was not evaluated using any measurement tool in the present study. However, the participants’ descriptive response, reflective reports, and responses during the focus group discussions suggest that the intervention could potentially have an extended impact that is worth exploring in future studies.

## Discussion

The objective of this study was to evaluate the effect of a peer-led intervention combining mental health promotion with group workshops on the mental health awareness and help-seeking behavior and to assess the impact of the intervention on the mental health state and well-being of university students in Hong Kong.

### Mental health awareness and prevention

Promotion of mental health enhances the ability of a person to reach and maintain a positive psychosocial state to cope with daily life adversities [5]. Efforts in promoting mental health include reducing stigmatization to prevent social exclusion and encouraging help-seeking behavior [28]. Mental health promotion is the first step to reduce stigmatization and improve mental health awareness, and it is often combined with other strategies [29]. In mental health promotion interventions, information is provided using presentations, discussions, stimulations or audio-visual aids targeting a specific population [29]. The message provided in the mental health promotion approach is crucial for the effect of stigma reduction [29]. The present study demonstrated that a peer-led intervention combining mental health promotion with coping-strategy-based group workshops increased the participants’ awareness of mental health.

Enhancing mental health awareness also includes providing knowledge to identify signs and symptoms of mental illness which is crucial for help-seeking, access to treatment and adoption of preventive measures [30, 31]. In the present study, participants were able to define mental health as a state of relaxation in which they can control their emotions, manage stress, and find balance. They also discriminate emotions linked to a good mental health state such as joy, happiness from the emotions linked to unbalanced mental health state such as fear, negative mood, stress, depression, loneliness, and hopelessness. Furthermore, participants mentioned situations that could be signs of emotional distress such as prolonged negative mood, social isolation, experiencing strong fear, and difficulties to be alone.

Not only the information provided in the mental health promotion part of the program helped the participants increase their knowledge on mental health and get familiarized with the signs and symptoms of mental health issues, but also the activities they participated in allowed them to carry out a self-evaluation regarding their emotional state. Through their own experience in the workshops, participants were able to identify their signs and symptoms of emotional distress, and the session served as an opportunity to practice self-awareness. Therefore, the intervention worked as an experiential strategy to stimulate self-awareness for the identification of signs and symptoms of emotional distress. When experiencing mental health issues, the help-seeking process starts with knowledge, recognition, awareness, and appraisal of symptoms [32]. In the present study, the mental health promotion combined with workshops, that have a positive impact on mental health, showed increased mental health awareness in the participants and stimulated self-awareness of the signs and symptoms that indicate emotional distress. Particularly, a peer-led program seems to have potential when it comes to supporting mental health among university students [33, 34]. For instance, Patalay et al., [34] found that a peer-designed and conducted mental health literacy program had high acceptability as 70% of the students agreed having enjoyed the program particularly that it was taught by university students. Furthermore, a study conducted by Byrom [33] on a peer support intervention for university students focused on mental health revealed a significant increase in mental wellbeing suggesting the potential of peer support.

### Benefits of the combined strategy

The intervention used in the present study showed to have a positive perceived impact identified as (a) increased knowledge and experiential identification of signs and symptoms of emotional distress, (b) knowledge of new coping strategies when facing emotional distress, and (c) identification of strategies to help others.

Insufficient education regarding mental health coping strategies is one of the biggest issues among university students [7]. Coping is a response that aims to diminish the physical, emotional and psychological burden related to stressful life events [35]. Often, students have minimal or maladaptive coping strategies when dealing with emotional distress [2, 4]. One of the hypotheses in the present study was that the workshops would allow participants to learn and experience ways to tackle emotional distress and manage their emotions. The benefits of the workshops reported by participants were attributed to the learned techniques as ways to release stress in case of emotional distress.

The participants’ experiences indicate that these new techniques could be considered as new coping strategies. Insufficient coping strategies have been identified as a risk factor for physical and mental health problems [2]. Previous research on general health of college students showed a correlation between stress-induced burden linked to reduced academic performance and lack of coping skills [2]. College students tend to adopt maladaptive coping behavior such as denial, self-blaming, and substance use which are main predictor of depression, anxiety, and stress [36]. Therefore, providing students with information on effective coping strategies such as healthy diet, regular physical activity, taking breaks, practicing relaxation techniques (e.g. yoga, meditation) is highly recommended [37].

Another benefit of the combined strategy refers to the use of the knowledge and skills learned to help others. Participants reported strategies to help others including listening, provide positive feedback and advice, offer social support, and advice-seeking professional help. Furthermore, participants used the information provided and their experience in the workshops to extend the benefit and help others who experience emotional distress. Taken all together, using a combined strategy including mental health promotion and workshops consisting of activities with reported benefit on mental health increased mental health awareness as observed in the quantitative analysis, students experienced new activities, and they found the activities helpful to release energy and negativity, and to get relaxed which reflects the positive impact of the intervention.

### Awareness of help‐seeking behavior

Poor mental health in young students between 15 and 24 years old is linked to the highly competitive Hong Kong education system and family pressure to achieve high academic goals [38, 39]. Hong Kong’ higher education student population has mild to severe depressive symptoms (68.5%) and anxiety (54.5%) [40]. In addition to the high academic pressure, the presence of stigma and discrimination associated with mental health issues in Hong Kong is high affecting their willingness to seek for help [39]. Knowledge of mental health increases self-awareness to identify emotions and causes of distress, reduces the stigma attached to mental illness and increases the willingness to seek help in case of emotional distress [12].

Although no statistically significant difference was observed in help-seeking behavior, participants showed increased awareness of the importance of help-seeking behavior when dealing with mental health challenges. For example, participants mentioned strategies to deal with mental health challenges such as communicating and sharing, self-care, being active, social support, and seek professional help.

## Limitations

One of the limitations of the present study was the lack of a long-term follow-up to assess the sustained effect of the intervention on mental health awareness and help-seeking behavior of the participants. Furthermore, a single group pre-post design does not allow concluding that observed changes are due to the intervention due to the lack of a control group. Provision of a control group without intervention in the study, however, may seem unethical given the potential benefits of the combined intervention. Another limitation of the present study is that the extended impact of the program was not measured. For instance, participants mentioned that they shared the information learned in the activities with family and friends. Therefore, exploring the extended impact of the intervention using measurement tools could be included in future studies.

## Conclusions

The peer-led intervention combining mental health promotion with group workshops provided a positive impact through increased mental health awareness and knowledge of coping strategies on self-help and helping others among university students. Further study could focus on the impact of the intervention when applied regularly throughout the entire academic year.

## Data Availability

The datasets used and/or analysed during the current study are available from the corresponding author on reasonable request.

## References

[1] Corrigan PW, Watson AC (2002). Understanding the impact of stigma on people with mental illness. World Psychiatr World Psychiatr Assoc.

[2] Mohr C, Braun S, Bridler R, Chmetz F, Delfino JP, Kluckner VJ (2014). Insufficient coping behavior under chronic stress and vulnerability to psychiatric disorders. Psychopathology.

[3] Farrer L, Gulliver A, Chan JKY, Batterham PJ, Reynolds J (2013). Technology-based interventions for mental health in tertiary students: systematic review. J Med Internet Res.

[4] Ratanasiripong P, China T, Toyama S (2018). Mental health and well-being of University Students in Okinawa. Educ Res Int.

[5] Min J-A, Lee C-U, Lee C (2013). Mental health promotion and illness prevention: a challenge for psychiatrists. Psychiatry Investig.

[6] Kobau R, Seligman MEP, Peterson C, Diener E, Zack MM, Chapman D (2011). Mental health promotion in public health: perspectives and strategies from positive psychology. Am J Public Health.

[7] Giamos D, Lee AYS, Suleiman A, Stuart H, Chen S-P (2017). Understanding Campus Culture and Student Coping Strategies for Mental Health Issues in Five Canadian Colleges and Universities. Can J High Educ.

[8] Eschenbeck H, Lehner L, Hofmann H, Bauer S, Becker K, Diestelkamp S (2019). School-based mental health promotion in children and adolescents with StresSOS using online or face-to-face interventions: study protocol for a randomized controlled trial within the ProHEAD Consortium. Trials.

[9] Gulliver A, Griffiths KM, Christensen H (2010). Perceived barriers and facilitators to mental health help-seeking in young people: a systematic review. BMC Psychiatry.

[10] Cornally N, McCarthy G (2011). Help-seeking behaviour: a concept analysis. Int J Nurs Pract.

[11] Kalra G, Christodoulou G, Jenkins R, Tsipas V, Christodoulou N, Lecic-Tosevski D (2011). Mental health promotion: guidance and strategies. Eur Psychiatry.

[12] Hui AK, Wong PW, Fu K (2014). Building a model for encouraging help-seeking for depression: a qualitative study in a Chinese society. BMC Psychol.

[13] Büssing A, Michalsen A, Khalsa SBS, Telles S, Sherman KJ, Sherman KJ (2012). Effects of yoga on mental and physical health: a short summary of reviews. Evid Based Complement Altern Med.

[14] Hölzel B, Ott U. Relationships between meditation depth, absorption, meditation practice, and mindfulness: a latent variable approach. J Transpers Psychol. 2006;38.

[15] Madhava R, Pulsifer C (2015). An impact of Jacobson’s Progressive Muscle Relaxation (JPMR) in managing the perceived stress level among College Students. Int J Res Med Appl Sci.

[16] Jacobson E (1938). Progressive relaxation.

[17] Kuyken W, Weare K, Ukoumunne OC, Vicary R, Motton N, Burnett R (2013). Effectiveness of the Mindfulness in Schools Programme: non-randomised controlled feasibility study. Br J Psychiatry.

[18] Seaward BL (2006). Managing stress: principles and strategies for health and wellbeing.

[19] Lauren Merianos A (2014). Vigorous physical activity among college students: using the health belief model to assess involvement and social support. Arch Exerc Heal Dis.

[20] Raglin JS (1990). Exercise and mental health: beneficial and detrimental effects. Sport Med.

[21] Ho FKW, Louie LHT, Hing-Sang Wong W, Chan KL, Tiwari A, Chow CB (2017). A sports-based youth development program, teen mental health, and physical fitness: An RCT. Pediatrics.

[22] Stathopoulou G, Powers MB, Berry AC, Smits JAJ, Otto MW (2006). Exercise interventions for mental health: a quantitative and qualitative review. Clin Psychol Sci Pract.

[23] Hanson WE, Creswell JW, Plano Clark VL, Petska KS, David Creswell J, Clark P (2005). Mixed methods research designs in counseling psychology. J Counsel Pshchol.

[24] Sandelowski M (2000). Combining Qualitative and Quantitative Sampling, Data Collection, and Analysis Techniques in Mixed-Method Studies. Res Nurs Health.

[25] Evans-Lacko S, Little K, Meltzer H, Rose D, Rhydderch D, Henderson C (2010). Development and psychometric properties of the Mental Health Knowledge Schedule. Can J Psychiatry.

[26] Fisher E, Amerigo F, Fischer EH, Farina A (1995). Attitudes toward seeking professional psychological help: a shortened form and considerations for research. J Coll Stud Dev.

[27] Braun V, Clarke V (2006). Using thematic analysis in psychology. Qual Res Psychol.

[28] Yamaguchi S, Mino Y, Uddin S (2011). Strategies and future attempts to reduce stigmatization and increase awareness of mental health problems among young people: a narrative review of educational interventions. Psychiatry Clin Neurosci.

[29] Heijnders M, Van Der Meij S (2006). The fight against stigma: an overview of stigma-reduction strategies and interventions. Psychol Health Med.

[30] Picco L, Seow E, Chua BY, Mahendran R, Verma S, Chong SA (2017). Recognition of mental disorders: findings from a cross-sectional study among medical students in Singapore. BMJ Open.

[31] Srivastava K, Chatterjee K, Bhat P (2016). Mental health awareness: The Indian scenario. Ind Psychiatry J.

[32] Gagnon MM, Gelinas BL, Friesen LN (2017). Mental Health Literacy in Emerging Adults in a University Setting: distinctions between symptom awareness and appraisal. J Adolesc Res.

[33] Byrom N (2018). An evaluation of a peer support intervention for student mental health. J Ment Health.

[34] Patalay P, Annis J, Sharpe H, Newman R, Main D, Ragunathan T (2017). A pre-post evaluation of OpenMinds: a Sustainable, Peer-Led Mental Health Literacy Programme in Universities and Secondary Schools. Prev Sci.

[35] Snyder CR, Ford CE, Harris RN. The Effects of Theoretical Perspective on the Analysis of Coping With Negative Life Events. In: Coping with Negat Life Events. Berlin: Springer; 1987. p. 3–13.

[36] Mahmoud JSR, Staten RT, Hall LA, Lennie TA (2012). The relationship among young adult college students’ depression, anxiety, stress, demographics, life satisfaction, and coping styles. Issues Ment Health Nurs.

[37] Negi AS, Khanna A, Aggarwal R (2019). Psychological health, stressors and coping mechanism of engineering students. Int J Adolesc Youth.

[38] Chan SM, Chan SK, Kwok WW (2014). Ruminative and catastrophizing cognitive styles mediate the association between daily hassles and high anxiety in Hong Kong adolescents. Child Psychiatry Hum Dev.

[39] Wong PWC, Arat G, Ambrose MR, Qiuyuan KX, Borschel M (2019). Evaluation of a mental health course for stigma reduction: a pilot study. Cogent Psychol.

[40] Lun KWC, Chan CK, Ip PKY, Ma SYK, Tsai WW, Wong CS (2018). Depression and anxiety among university students in Hong Kong. Hong Kong Med J.

